# Pretargeted Nuclear Imaging and Radioimmunotherapy Based on the Inverse Electron-Demand Diels–Alder Reaction and Key Factors in the Pretargeted Synthetic Design

**DOI:** 10.1155/2019/9182476

**Published:** 2019-08-25

**Authors:** Lin Qiu, Wujian Mao, Hongyan Yin, Hui Tan, Dengfeng Cheng, Hongcheng Shi

**Affiliations:** Department of Nuclear Medicine, Zhongshan Hospital, Fudan University, Shanghai 200032, China

## Abstract

The exceptional speed and biorthogonality of the inverse electron-demand Diels–Alder (IEDDA) click chemistry between 1,2,4,5-tetrazines and strained alkene dienophiles have made it promising in the realm of pretargeted imaging and therapy. During the past 10 years, the IEDDA-pretargeted strategies have been tested and have already proven capable of producing images with high tumor-to-background ratios and improving therapeutic effect. This review will focus on recent applications of click chemistry ligations in the pretargeted imaging studies of single photon emission computed tomography (SPECT), positron emission tomography (PET), and pretargeted radioimmunotherapy investigations. Additionally, the influence factors of stability, reactivity, and pharmacokinetic properties of TCO tag modified immunoconjugates and radiolabeled Tz derivatives were also summarized in this article, which should be carefully considered in the system design in order to develop a successful pretargeted methodology. We hope that this review will not only equip readers with a knowledge of pretargeted methodology based on IEDDA click chemistry but also inspire synthetic chemists and radiochemists to develop pretargeted radiopharmaceutical components in a more innovative way with various influence factors considered.

## 1. Introduction

Since its advent over a decade and a half ago, click chemistry has been used in nearly all disciplines of modern chemistry, including drug discovery, bioconjugation, materials science, nanoscience, and radiochemistry [1]. However, these previous generations of click reactions are not without their limitations. For example, the requirement of a metal catalyst in Cu(I)-catalyzed 1,3-dipolar cycloaddition between azides and alkynes (CuAAC) can be a complication when used in conjunction with radiometals. In contrast, the hydrophobicity and cumbersome synthesis of the cyclooctyne precursors in the strain-promoted azide-alkyne cycloaddition (SPAAC) have proven limiting to their widespread application. Additionally, the somewhat sluggish kinetics of the SPAAC system almost certainly precludes its use for *in vivo* pretargeted imaging or therapy [2]. In response to these limitations, the past 10 years have witnessed the rise of a more promising click ligation: the inverse electron-demand [4 + 2] Diels–Alder (IEDDA) cycloaddition between a 1,2,4,5-tetrazine (Tz) and a strained alkene dienophile. The IEDDA reaction is efficient, rapid, modular, bioorthogonal, and compatible with aqueous environments and proceeds without a catalyst. But what really sets it apart from other click ligations is its speed. Rate constants for the reaction between Tz dienes and trans-cyclooctene (TCO) dienophiles can exceed 100,000 M/s. The potential of the IEDDA reaction as a tool for bioconjugation was recognized almost immediately and has been proven to be highly effective *in vivo* for a wide range of applications [3–5]. Monoclonal antibodies (mAbs) have been used for many years to deliver radionuclides to targeted tissues due to their exquisite affinity and selectivity for molecular targets. However, slow pharmacokinetics of mAb necessitates radiolabelling using radionuclides with moderate and long half-lives, which creates prohibitively high radiation dose to healthy organs [6, 7]. Pretargeted methodology was designed to avoid the high radiation exposure due to the slow pharmacokinetics of radioimmunoconjugates and high background doses by decoupling the antibody from the radioisotope and injecting the two components separately [8]. The pretargeted approach consists of two steps. First, target-specific immunoconjugates are injected and bind to the target site and clear slowly. Next, radiolabeled compounds are added, which selectively react with the immunoconjugates bound to the target and clear rapidly. This pretargeted method presents several advantages, including superior image contrast, a decrease in the radiation doses to the nontarget organs [8], and possible use of short-lived radionuclides that would normally be incompatible with antibody-based vectors [9].

The pretargeted approach requires a rapid and selective chemical reaction in *in vivo* models. These two traits are hallmarks of the IEDDA ligation. Devaraj et al. [10, 11] and Jewett et al. [12] first applied the bioorthogonal chemical reaction to pretargeted live cell imaging. The pioneering works paved a way for nuclear medicine application based on bioorthogonal IEDDA click reaction. Currently, the IEDDA click reaction had been applied in pretargeted nuclear imaging and radioimmunotherapy and showed a promising prospective [13–35].

In this review, we provided a brief introduction about these investigations of pretargeted nuclear imaging and radioimmunotherapy based on IEDDA click reaction. Additionally, for the development of a successful pretargeted methodology, several components should be carefully considered in the system design: antibody, tetrazine, dienophile, chelator, radionuclide, linker, or other modifications. The influence factors of stability, reactivity, and pharmacokinetic properties of TCO tag modified immunoconjugates and radiolabeled Tz-derivatives were also summarized in this article, which should be taken into consideration in the synthetic design of pretargeted methodology based on IEDDA click reaction.

## 2. IEDDA Click Chemistry in Pretargeted Nuclear Imaging and Radioimmunotherapy: A Brief Historical Summary

### 2.1. Pretargeted Nuclear Imaging with SPECT

The first successful application of pretargeted nuclear imaging based on IEDDA click chemistry with SPECT was reported by Rossin et al. [13] in 2010. In this work, a noninternalizing TCO-modified mAb, targeting the tumor-associated glycoprotein 72 (TAG-72), was administrated to mice bearing LS174T xenografts (Table 1). After a lag time of 24 h, a DOTA-functionalized bispyridyl Tz labeled with indium-111 (^111^In-DOTA-PEG_11_-Tz) was administered. Three hours after injection of ^111^In-tetrazine, SPECT imaging clearly delineated the tumor with a tumor uptake quantification of 4.2% ID/g and a tumor-to-muscle ratio of 13.1. Blood and the liver revealed low levels of radioactivity due to residual circulating CC49-TCO. As negative controls, mice were injected with either CC49 without TCO or nonspecificity TCO-modified rituximab for TAG-72. In both cases, a significantly reduced radioactivity was accumulated in the tumors that could not be discriminated from the surrounding tissue.

The first promising results encouraged Rossin et al. to further optimize the pretargeted approach in subsequent studies. The authors improved the *in vivo* reactivity of the TCO tag and demonstrated that the reaction constant is up to 10-fold higher for substitution of TCO in the axial position rather than the equatorial position with bulky linkers [14]. In the previous work, the TCO attached to the mAb CC49 was slowly deactivated by cis-trans isomerization due to a long linker length [-O-CH_2_-C_6_H_4_-C(O)-NH-(CH_2_)_2_-PEG_12_-C(O)-O-] [13]. By removing the NH-(CH_2_)_2_-PEG_12_-C(O) linker, Rossin et al. reported that a shorter linker length [-O-CH_2_-C_6_H_4_-C(O)-O-] between CC49 and the TCO tag improved the TCO stability dramatically, leading to a higher target accumulation and improved imaging contrast [14, 15]. Rossin and colleagues furthermore developed a clearing agent capable of eliminating circulating CC49-TCO from the blood to the liver, prior to tetrazine probe injection. This setup resulted in more than a 100-fold higher imaging contrast compared to the results from the original study [15]. In a more recent study, the authors optimized the pharmacokinetics of the tagged antibody by using a less hydrophobic acetamide linker between CC49 and TCO compared with a benzamide linker. The longer blood clearance half-life of the TCO-modified CC49 resulted in an increased tumor uptake of the small and fast clearing radiolabeled tetrazine probe and increased tumor-to-nontumor ratios [16].

The small size of affibody molecules is favorable for rapid localization in tumors and clearance of unbound tracer from circulation, which provides high tumor-to-background ratio during imaging. However, many radiometal-labeled affibody molecules have high renal reabsorption [17–19]. A possible way to solve the issue of high renal reabsorption of radiometal-labeled affibody molecules is the application of pretargeted methodology. Altai et al. [20] conjugated TCO to the anti-human epidermal growth factor receptor 2 (HER2) affibody molecule Z_2395_ (TCO-PEG_4_-Z_2395_). DOTA tetrazine was labeled with ^111^In (^111^In-DOTA-PEG_10_-Tz). Subsequently, *in vivo* pretargeted biodistribution showed the tumor uptake of ^111^In-tetrazine was approximately 2-fold higher than the renal uptake. Pretargeting provided a 56-fold reduction of renal uptake in comparison with direct targeting (5 ± 2% ID/g vs. 284 ± 22%ID/g). The micro-SPECT images also demonstrated the difference clearly. Similarly, in order to reduce the renal reabsorption and nephrotoxicity of radiometal-labeled peptides or antibody fragments, van Duijnhoven et al. [21] developed a pretargeted strategy between TCO-functionalized AVP04-07 diabodies (AVP04-07-TCO) and a radiolabeled tetrazine probe (^111^In/^177^Lu-DOTA-PEG_11_-Tz). Pretargeted AVP04-07-TCO/^111^In-DOTA-PEG_11_-Tz biodistribution and SPECT/CT image studies revealed the tumor-to-kidney ratio was more than 20-fold higher than that previously reported with a radiolabeled DOTA-conjugated AVP04-07 diabody [22], indicating that pretargeted strategy could significantly reduce the kidney radiation dose of diabody.

^99m^Tc is the most widely used radionuclide for diagnosis in nuclear medicine with a suitable emission of 140 keV and 6-hour half-life. García et al. [23] developed a pretargeted approach using the CC49-TCO/^99m^Tc-HYNIC-Tz combination in order to expand the application and utility of ^99m^Tc-labeled antibodies. A biodistribution study with pretargeted CC49-TCO/^99m^Tc-HYNIC-Tz showed a modest amount of tumor uptake at 1.39 ± 0.43% ID/g, which was consistent with the imaging study. The majority of the radioactivity was in the liver and gastrointestinal tract and the kidneys. However, tumor uptake of ^125^I-CC49-TCO was high at 43.53%ID/g. The authors concluded that the low tumor ^99m^Tc-HYNIC-Tz uptake was not due to a lack of CC49-TCO but likely the result of being sequestered in the liver and gastrointestinal tract and unavailable for reaction with tumor targeted CC49-TCO. The biodistribution and imaging studies indicated the need of a more hydrophilic ^99m^Tc-HYNIC-Tz derivative.

In order to increase the hydrophilicity of the ^99m^Tc-HYNIC-Tz conjugate and improve its *in vivo* pharmacokinetic properties, subsequently, García et al. [24] explored the incorporation of a polyethylene glycol spacer (^99m^Tc-HYNIC-PEG_4_-Tz) and/or a charged amino acid polypeptide sequence (^99m^Tc-HYNIC-PEG_5_-polypeptide-Tz) between the HYNIC and Tz. Both compounds cleared rapidly from blood, exhibiting 0.95 ± 0.40% ID/g and 2.23 ± 0.45% ID/g in blood at 1 h after injection, respectively. The polypeptide-conjugated Tz derivative showed reduced gastrointestinal accumulation and increased kidney clearance, with 81.92 ± 5.06% IDs eliminated by urine after 1 h. The increased hydrophilic character could be attributable to potential molecular polypeptide sequence and charge of the compound.

By adding the bisphosphonate (BP) to TCO to create a bisphosphonate-modified variant of TCO (TCO-BP), Yazdani et al. developed a bone-seeking pretargeted and bioorthogonal strategy. TCO-BP represents an effective ligand for delivering trans-cyclooctene to sites of active bone remodeling without having to use antibodies. This approach can be used for the delivery of diagnostic radioisotope (^99m^Tc-Tz complex) to the bone. The ^99m^Tc-labeled derivative/TCO-BP pretargeted method demonstrated selective localization to shoulder and knee joints in a normal mice biodistribution study [25].

### 2.2. Pretargeted Nuclear Imaging with PET

**Figure 1:**
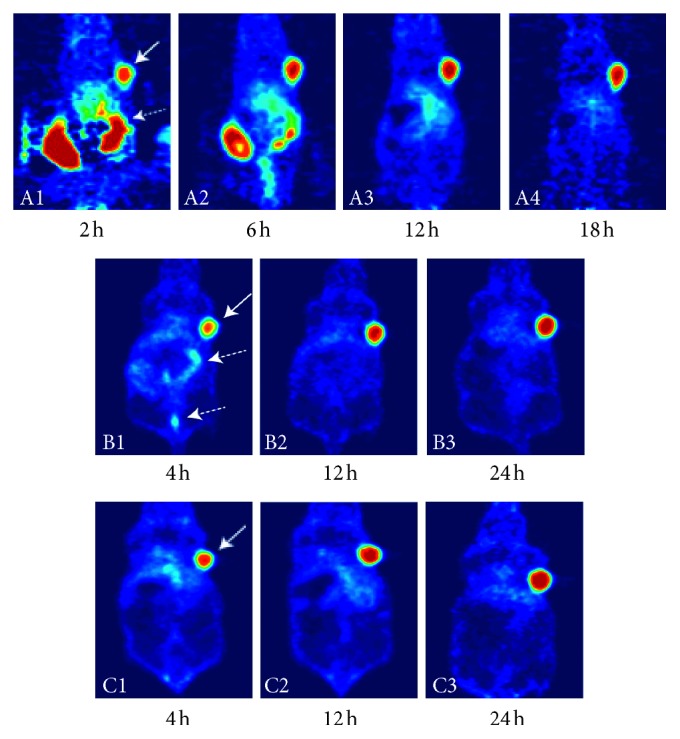
Pretargeted PET imaging comparison of A33-TCO/^64^Cu-NOTA-Tz (A1-4), A33-TCO/^64^Cu-NOTA-PEG_7_-Tz (B1-3), and A33-TCO/^64^Cu-SarAr-Tz (C1-3). Mice bearing subcutaneous SW1222 xenografts were administered A33-TCO via tail vein injection. After 24 h, the same mice were administered ^64^Cu-NOTA-Tz, ^64^Cu-NOTA-PEG_7_-Tz, and ^64^Cu-SarAr-Tz, respectively. All three ^64^Cu-labeled Tz radioligands were successfully applied to pretargeted experiments and delineated the tumor clearly (solid white arrows). ^64^Cu-NOTA-Tz was eliminated slowly through the gastrointestinal pathway (dashed white arrows). ^64^Cu-SarAr-Tz cleared quickly and primarily through the urinary tract, and ^64^Cu-NOTA-PEG_7_-Tz represented an intermediate case with excretion through both the gastrointestinal and urinary tracts (dashed white arrows) [26, 27].

Zeglis et al. [26] extended the *in vivo* pretargeted click methodology from SPECT to PET. In pretargeted experiments, nude mice with subcutaneous SW1222 xenografts were intravenously administrated with 100 *μ*g huA33-TCO, followed 24 h later by ^64^Cu-NOTA-Tz (10.2–12.0 MBq). Radioactivity accumulation in the tumor was approximately 4.1% ID/g one hour after ^64^Cu-NOTA-tetrazine injection. Interestingly, although traditional imaging with directly ^64^Cu- or ^89^Zr-labeled huA33 showed higher tumor uptake than the pretargeted huA33, the pretargeted approach yielded comparable images and significantly higher tumor-to-muscle ratios. Unfortunately, the rather lipophilic tetrazine applied in this study showed mainly hepatobiliary excretion with high activity in the gastrointestinal tract up to 12 hours, which is not optimal when imaging colon cancer. In subsequent investigation, Zeglis group [27] designed two novel ^64^Cu-labeled Tz radioligands to increase hydrophilicity and obtain renal clearance. The first, ^64^Cu-Tz-PEG_7_-NOTA, was designed with a PEG_7_ spacer separating the Tz scaffold from the NOTA chelator. For the second, ^64^Cu-SarAr-Tz, a sarcophagine-based chelator, replaced the NOTA macrocycle, which changed the overall charge of the Tz radioligand from −1 to +2. The newly designed ^64^Cu-SarAr-Tz clears quickly and primarily through the urinary tract, and ^64^Cu-NOTA-PEG_7_-Tz represents an intermediate case with excretion through both the intestines and the kidneys (Figure 1). Despite different pharmacokinetic profiles, both two ^64^Cu-labeled Tz radioligands were successfully applied to pretargeted experiments, with ^64^Cu-SarAr-Tz showing a relatively higher % ID/g in huA33-TCO/^64^Cu-labeled-Tz pretargeted biodistribution experiment.

The advantages of pretargeted imaging (i.e., maximizing imaging contrast while reducing radiation exposure to healthy tissue) could be exploited more efficiently by using shorter-lived radionuclides such as ^68^Ga and ^18^F, compared to medium-lived radionuclides such as ^64^Cu, ^111^In, and ^99m^Tc. In 2014, Evans et al. [28] reported the radiosynthesis of ^68^Ga-DOTA-Tz and its evaluation in pretargeted experiments with cetuximab-PEG_4_-TCO as the primary targeting agent. *In vivo* pretargeted biodistribution in A431-xenograft bearing mice was performed and resulted in a tumor uptake of 3.48% ID/mL. However, the PET image analysis indicated high retention of the radioactivity in the liver. The authors suggested that the high level of activity in the liver was characteristic with the pharmacokinetic profile of mAb because high liver uptake was also observed in the biodistribution of directly radiolabeled cetuximab. A high TCO-loading/mAb ratio of 17 : 1 might be another reason of high hepatic uptake.

Nichols et al. [5] reported a ^68^Ga-labeled Tz-coated polymer based on an aminodextran backbone. The polymer scaffold was chosen due to its well-established clinical safety record, hydrophilicity, low cost, ready availability in numerous molecular weights, and the author's previous experience working with dextran imaging agents [29, 30]. The 16 kDa polymer was functionalized with the chelator DTPA before amide was coupled to an NHS-functionalized Tz. Pretargeted PET imaging demonstrated the ability of ^68^Ga tetrazine dextran to react with the A33-TCO modified antibody preinjected as the primary agent. The tumor-to-muscle ratio was 3.9 ± 1.8% ID/g in biodistribution experiment, and a prolonged retention in blood and a high liver uptake was observed, possibly due to partial release of ^68^Ga from DTPA *in vivo* [31]. Devaraj et al. [29] also reported a Tz-coated polymer radiolabeled with fluorine-18 (^18^F) based on the same aminodextran backbone. In this investigation, polymer-modified tetrazine with a 10 kDa dextran (PMT10) was synthesized and then functionalized with monosubstituted Tz derivatives. Radiolabeling was achieved by indirect ^18^F-fluorination in a decay-corrected radiochemical yield of 89.2%.

Considering the well-documented instability of tetrazines under the alkaline conditions required for nucleophilic ^18^F-fluorination reactions, the Al[^18^F]-labeling approach seems to be particularly appropriate for the synthesis of ^18^F-labeled Tz-derivative radioligands [32, 33]. In 2016, Meyer et al. [34] reported the first Al[^18^F]-NOTA labeling of a Tz scaffold (Al[^18^F]-NOTA-PEG_11_-Tz). Both pretargeted 5B1-TCO/Al[^18^F]-NOTA-PEG_11_-Tz PET image and biodistribution showed that the tumor uptake increased with time (from 3.0 ± 0.32% ID/g at 30 min to 5.6 ± 0.85% ID/g at 4 h), while the activity concentrations in the blood and intestines concomitantly decreased. The plasma half-life of the Al[^18^F]-NOTA-PEG_11_-Tz was 71.2 ± 5.40 min. The rather long half-life might be an explanation to the increasing tumor uptake over time.

In almost all reports of IEDDA-based pretargeted investigations, TCO was attached to the antibody and the tetrazine formed part of the radioligand. However, Shi et al. applied an inverse strategy and replaced TCO with a Reppe anhydride derivative. In this work, two EGFR-specific monoclonal antibodies was modified by tetrazine (cetuximab-Tz and panitumumab-Tz) and Reppe anhydride derivative was radiolabeled using Al[^18^F]-NOTA (Al[^18^F]-NOTA-TD). Small animal PET/CT quantification analysis showed a high radioactivity in the tumor of nude mice pretreated with cetuximab-Tz (6.33 ± 0.71% ID/g) and panitumumab-Tz (8.73 ± 1.04% ID/g). The tumor-to-muscle ratios were 7.12 ± 1.23 and 8.97 ± 1.82, respectively [35].

**Figure 2:**
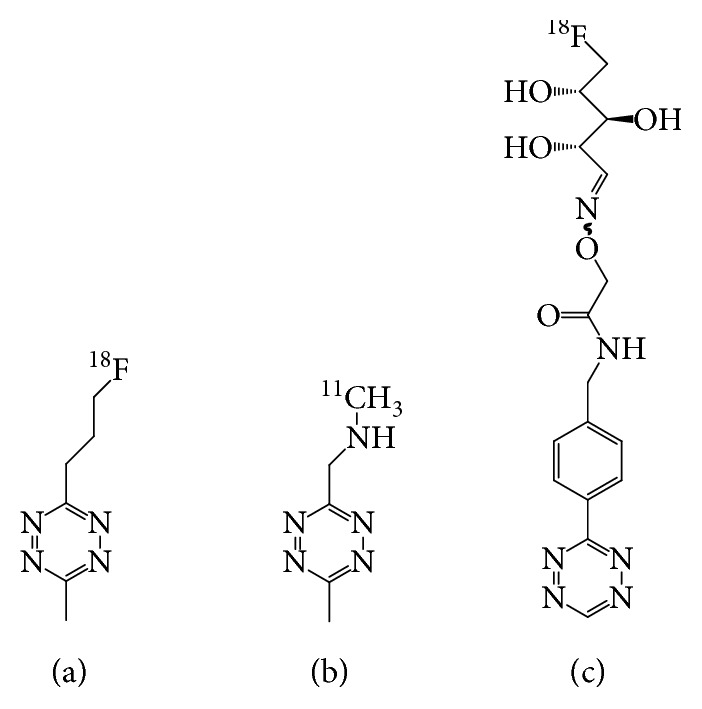
Structures of the small molecular ^11^C- and ^18^F-labeled tetrazines [36, 42, 43].

It is known that chelator-based structures cannot cross cell membranes and many mAbs internalize to some degree, which limit or even in some cases prevent the use of radiometal-labeled Tz derivatives. Thus, efforts have been made toward developing Tz scaffolds labeled with carbon-11 and fluorine-18 directly. These compounds would have the potential to be used for intracellular targets. Many investigators have reported various Tz derivatives labeled with ^11^C and ^18^F although direct labeling with these radionuclides requires harsh reaction conditions [36–38, 42, 43]. However, until recently, no pretargeted imaging experiments were reported. In this work conducted by Denk et al. [42], a water-soluble ^18^F-labeled tetrazine (Figure 2(a)) using direct ^18^F-fluorination was developed, with fast and reproducible pharmacokinetics. In addition, the ligation to a TCO was detected *in vivo* and radio-TLC analysis showed more than 90% conversion to the ligation adduct in only 5 min. This was a promising result for future evaluation in pretargeted imaging studies. In 2016, Denk et al. [36] designed and synthesized a ^11^C-labeled Tz (Figure 2(b)) for pretargeted PET imaging. In the pretargeted experiment, TCO- or s-TCO-modified mesoporous silica nanoparticles (MSNs) were administered to female BALB/c mice first. After sufficient time for accumulation of the MSNs-TCO or MSNs-s-TCO in the lung (5 min), ^11^C-labeled Tz was injected and dynamic PET scanning was conducted. A significantly increased activity concentration in the lung was observed.

Keinänen et al. [43] synthesized a glycosylated ^18^F-labeled Tz (Figure 2(c)) with high yield, purity, and specific activity under mild reaction conditions via conjugation with 5-[^18^F]fluoro-5-deoxyribose. Subsequently, Keinänen et al. [37] reported a successful pretargeted PET imaging based on the ^18^F-labeled Tz and TCO-modified silicon nanoparticles (NPs-TCO). The fast IEDDA reaction resulted in high radioactivity accumulation in the expected organs (spleen and liver) within 10 min after the administration of the ^18^F-Tz. Using the same ^18^F-labeled Tz as a radiotracer, Keinänen et al. [38] investigated pretargeted methodology for tracing two clinically relevant, internalizing mAbs, TCO-modified cetuximab and trastuzumab. For both antibodies, PET images demonstrated the tumor could be clearly visualized with the highest uptake of 3.7 ± 0.1% ID/g for cetuximab and 1.5 ± 0.1% ID/g for trastuzumab as quantified by *ex vivo* biodistribution.

### 2.3. Pretargeted Radioimmunotherapy

The remarkable affinity of antibodies for tumor biomarkers made them attractive vectors for the selective delivery of therapeutic radionuclides to cancer cells. However, in radioimmunotherapy (RIT), the use of therapeutic isotopes with long physical half-lives was mandated due to the slow pharmacokinetics of mAbs, which can potentially result in prohibitively high radiation doses to healthy organs, particularly the bone marrow and kidneys. Pretargeted RIT (PRIT) is a promising approach for the delivery of a therapeutic radiation dose to solid tumors while sparing normal tissues by decoupling the targeting vector from the radioisotope. Currently, several therapeutic radionuclide-labeled Tz frameworks based on the pretargeted click chemistry have been developed and evaluated *in vivo* [22, 25, 39–41, 44]. However, only two of these Tz-derivatives have been reported to be evaluated for therapeutic efficacy using PRIT as follows.

**Figure 3:**
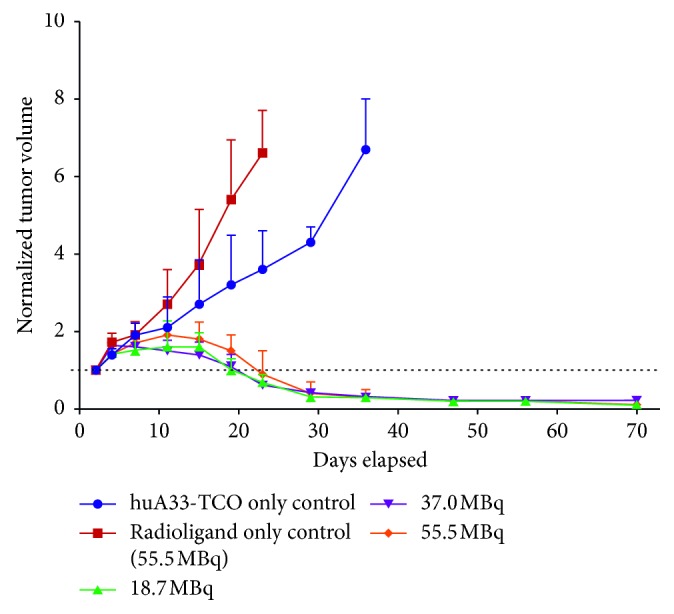
Normalized tumor volume for each group of mice in the pretargeted huA33-TCO/^177^Lu-DOTA-PEG_7_-Tz radioimmunotherapy study. The two control groups received either huA33-TCO (blue) or Tz radioligand (red) only. The three treatment groups received huA33-TCO followed 24 h later by 18.5 (green), 37.0 (purple), or 55.5 (orange) MBq of ^177^Lu-DOTA-PEG_7_-Tz. Striking differences were observed between treatment and control groups [40].

In 2017, Houghton and colleagues [39] synthesized two novel ^177^Lu-labeled tetrazine-bearing radioligands (^177^Lu-DOTA-PEG_7_-Tz and ^177^Lu-CHX-A″-DTPA-PEG_7_-Tz) for PRIT. TCO-modified 5B1 was administrated in murine models of pancreatic cancer, and 72 h later, varying amounts of ^177^Lu-DOTA-PEG_7_-Tz (400, 800, and 1,200 *μ*Ci) were used in therapy groups. The control cohorts that had not been previously administered 5B1-TCO were injected with either 0.9% sterile saline or 1,200 *μ*Ci of ^177^Lu-DOTA-PEG_7_-Tz. *Ex vivo* biodistribution showed rapid (4.6 ± 0.8% ID/g at 4 hours) and persistent (16.8 ± 3.9% ID/g at 120 hours) uptake in tumors while concurrently clearing from blood and nontarget tissues in therapy groups. Tumor regression or growth delay was observed at higher doses when compared to control groups (Figure 3). Membreno et al. [40] also reported an investigation of a PRIT strategy based on the same Tz radioligand (^177^Lu-DOTA-PEG_7_-Tz) and huA33-TCO for colorectal carcinoma radiotherapy. The therapy study revealed striking differences between both the survival and tumor growth of the treatment and control cohorts.

Another PRIT study was reported by Shah et al [41]. In this study, four groups of LS174T tumor-bearing animals were injected with 100 *μ*g of CC49-TCO, followed by two doses of clearing agent at 30 and 48 h. Then, mice were treated with various doses (0, 2.78, 4.63, 7.40, and 2 × 2.78 MBq) of ^212^Pb-DOTA-PEG_11_-Tz. Two nontreated control groups of mice were injected with 100 *μ*L of PBS and 100 *μ*g of CC49-TCO without ^212^Pb-DOTA-PEG_11_-Tz. As a nonspecific control, a group of mice was injected with a TCO-functionalized mAb (RTX-TCO) lacking affinity and received 7.4 MBq of ^212^Pb-DOTA-PEG_11_-Tz. Although biodistribution revealed a low tumor uptake of 0.94% ID/g at 3 h p.i. and 0.66% ID/g at 24 h p.i., all the mouse groups receiving treatment displayed a dose-dependent reduction in tumor size. Mice that were administered with only PBS or just CC49-TCO did not have any reduction in the tumor growth rate. Mice in a nonspecific control exhibited slower tumor growth rate than that of the nontreated control groups but faster than the mice in the PRIT regimen. Unfortunately, high kidney uptake (2.5% ID/g 3 h p.i.) was observed, which prevented clinical translation of ^212^Pb-DOTA-PEG_11_-Tz.

## 3. Stability and Reactivity of Tetrazine and Dienophile

In almost all reported IEDDA-based pretargeted investigation, the TCO moiety was attached to the primary agent and the Tz formed part of the radioligand, which was mainly due to their differences in *in vivo* stability. The logical approach would be to functionalize the longer circulating primary agent with the relatively more stable TCO and use the less stable Tz for the faster cleared secondary agent. The stability and reactivity of both Tz derivative and TCO counterpart should be deliberately considered in pretargeted study design.

### 3.1. Stability and Reactivity of Tetrazine

**Figure 4:**
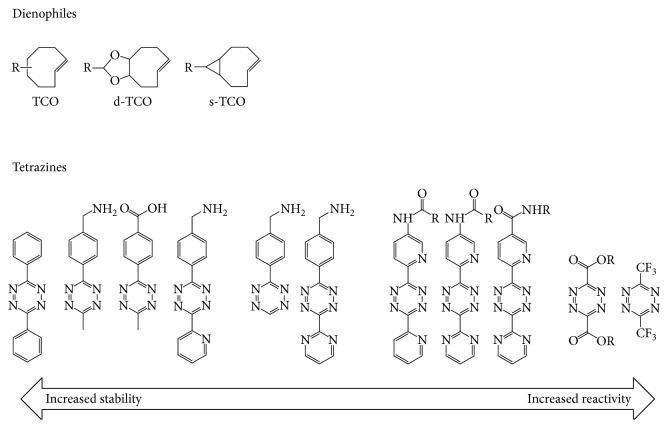
Stability and reactivity of various tetrazines and dienophiles [45].

One of the chief advantages of the IEDDA cycloaddition lies in its excellent speed. The rapidity of the reaction is governed by the identity of the tetrazine and dienophile. Several different tetrazines have also been tested for their kinetics, and the second-order reaction rate ranged from 210 M/s to almost 30,000 M/s in the cycloaddition reaction. An inverse correlation between stability and reaction rate was observed: a more stable tetrazine generally reacted less quickly, whereas a less stable compound generally reacted more rapidly [45, 46] (Figure 4).

Several authors investigated the stability of radiolabeled Tz-derivatives in *in vitro* assays. Zeglis et al. [27] reported that both ^64^Cu-Tz-PEG_7_-NOTA and ^64^Cu-Tz-SarAr were very stable in PBS (pH 7.4). However, more extensive decomposition was observed in human serum at later time points. For example, 64.0 ± 6.5% and 67.7 ± 4.3% of ^64^Cu-Tz-PEG_7_-NOTA and ^64^Cu-Tz-SarAr, respectively, remained intact after incubation for 8 h. Neither the release of ^64^Cu^2+^ from the chelators nor the binding of significant amounts of the radioligands to serum proteins was observed in these catabolites. These results are generally consistent with those of ^64^Cu-NOTA-Tz reported previously [26]. The authors suggested that the decomposition was related primarily to the breakdown of the tetrazine as all three constructs bear identical tetrazine moieties. In another Tz-scaffold bearing pyridyl substituents in the 3,6 positions (^111^In-DOTA-PEG_11_-Tz), Rossin et al. [13] reported similar results of the stability *in vitro* assays in PBS, serum, and blood. It is known that tetrazines are unstable, particularly under alkaline conditions; while the serum is of neutral pH, the additional presence of potentially nucleophilic sulfhydryl and amino groups in the serum may accelerate decomposition even at a neutral pH. However, given the exceptional speed of the IEDDA reaction with TCO, it was deemed unlikely that the decomposition of the tetrazines at later time points will severely impair their chances of functioning *in vivo* [26, 27].

### 3.2. Stability and Reactivity of Dienophile

For the dienophile, the norbornene derivatives were used as the dienophile in the early IEDDA reaction with a rate constant around 1-2 M/s in water at 37°C. However, norbornenes have quickly given way to dienophiles based on TCO, which were found to dramatically accelerate the reaction by over 3 orders of magnitude. Currently, the most frequently used dienophiles are based on TCO. TCO ring strain appears to have the greatest influence on its reactivity. However, similar to tetrazine, extremely high reactivity comes at the expense of *in vivo* stability (Figure 4). For example, the most reactive s-TCO derivative exhibiting a 16 hours *in vivo* stability half-life due to its isomerization to less stable cis-isomer over time [14].

To improve the *in vivo* reactivity and stability of the TCO tag, Rossin et al. [14] investigated the deactivation mechanism of mAb-TCO. The authors ruled out the possibility that the tag deactivation was due to cleavage of the tag from the antibody because the conjugate remained intact for 48 h in serum. Finally, Rossin group found the isomerization conversion from TCO into cis-cyclooctene (CCO) via copper-containing proteins (e.g., transcuprein, mouse serum albumin, and ceruloplasmin) was the sole deactivation pathway in serum. CCO is of 5 orders of magnitude less reactive toward tetrazines than TCO. The cis-isomerization can be significantly impeded by increasing the steric hindrance around the TCO tag. In that respect, the shorter acetamide linker compared with the benzamide linker or removal of the PEG linker between the TCO and mAb may have further increased the steric hindrance, thus hampering interaction with serum protein-bound copper. In addition, as albumin has binding pockets for hydrophobic TCO tags [47], the reduced hydrophobicity of the acetamide linker may have contributed to a reduced albumin interaction and corresponding TCO to CCO conversion. Biological half-life of up to 6 days was reached using a shorter linker between the TCO and mAb. In contrast, free TCO in mouse serum was completely isomerized to CCO within 8 h. Besides, TCO tag that was linked to the antibody through an axial substituent rather than the equatorial position with bulky linkers showed a 10-fold reactivity increase. Additionally, it is known that the isomerization of TCO to CCO can be induced thermally [48], by light [49], or in concentrated thiol solutions [50].

## 4. Influence Factors of Pharmacokinetic Properties of mAb-TCO and Radiolabeled Tz-Derivatives

### 4.1. Influence Factors of Pharmacokinetic Properties of mAb-TCO

#### 4.1.1. mAb-TCO Internalization

The first step in the development of the pretargeted methodology was the system design. Five components needed to be chosen: antibody, tetrazine, dienophile, radionuclide, and chelator. For the pretargeting to be successful, the antibody target, e.g., transmembrane cell-surface receptor, should be available for binding of the targeting antibody and the antibody-receptor complex should remain on the cell surface. The choice of antigen-antibody is a precondition for the development of a successful pretargeted methodology: the internalization and consequent sequestration of the mAb-TCO before the administration of the radiolabed Tz derivative would severely reduce the likelihood of *in vivo* click ligations. For antigen-targeting component that underwent internalization, the rate and extent of internalization should be taken into consideration to make a pretargeted system workable.

For example, the A33 antigen has been shown to exhibit cell surface persistence because of its association with tight junction proteins. The corresponding targeting antibody, A33, was chosen in several investigations and micro-PET/CT demonstrated high tumor uptake [26, 27, 29]. However, in the pretargeted study conducted by Keinänen et al., the tumor uptakes observed for both cetuximab-TCO and trastuzumab-TCO were much lower than the uptakes in their corresponding conventional imaging experiments radiolabeled with zirconium-89 directly. The reason for the low levels of radioactivity accumulation in the tumors for the pretargeted experiments might be explained by the fact that radiolabeled Tz can only react with the small number of mAbs that have not yet internalized [38]. Interestingly, Houghton et al. found that BxPC3 and Capan-2 cells internalized antibody 5B1, but Capan-2 did so more rapidly and to a greater extent [51]. Differences in the rate of internalization have also been observed with cell lines and other antibodies [52], but it remains unclear what contributes to the difference in kinetics and rate of internalization.

#### 4.1.2. TCO Tags of mAb

It has been demonstrated that conjugation of drugs to antibodies results in modification of antibody pharmacokinetics. For example, antibody-drug conjugates (ADCs) with relatively hydrophobic drug combinations clear faster from circulation than ADCs with more hydrophilic moieties [53]. Rossin et al. measured the blood kinetics of CC49-oxymethylbenzamide-TCO and CC49-oxymethylacetamide-TCO and observed a 1.6-fold increased blood clearance half-life of 22 h for CC49-oxymethylacetamide-TCO compared with the 14.1 h observed for CC49-oxymethylbenzamide-TCO [16]. Without considering other influencing factors, the relatively high protein surface hydrophobicity of the CC49-oxymethylbenzamide-TCO clears faster from circulation and thus may affect the pretargeted efficacy, leading to a lower tumor exposure and uptake.

For the modification of mAb with TCO, an average of less than 10 TCO moieties per antibody was reported by most investigators [13, 16, 21]. Low ratio of TCO/mAb has a limited effect on the lipophilicity and pharmacokinetic of mAb, and the hepatic uptake of pretargeted imaging was low in these investigations. However, in the pretargeted experiment performed by Evans et al. [28], an average of 17 TCO moieties had been added to each molecule of cetuximab. High hepatic uptake was observed in pretargeted biodistribution and imaging, which might be a consequence of the high TCO-loading/mAb.

#### 4.1.3. mAb-TCO Clearing Agents

The pharmacokinetics of mAb-TCO could be further improved by tetrazine-functionalized clearing agents. To minimize mAb-TCO retention in nontumor tissues, Rossin et al. [15] and Shah et al. [41] had developed a mAb-TCO clearing agent that could react with and rapidly remove residual mAb-TCO from the blood stream prior to injection of the radiolabeled tetrazine. In Rossin's study, a clearing agent of galactose-albumin construct functionalized with Tz derivative was applied in the CC49-TCO/^111^In-DOTA-PEG_11_-Tz pretargeted strategy. The results revealed that the blood level of CC49-TCO was lowest after the double dose (30 h and 48 h after mAb injection) of the clearing agent (0.19 ± 0.04% ID/g), followed by the single dose (30 h after mAb injection, 1.16 ± 0.43% ID/g) and highest in the group without clearing agent injection (8.47 ± 4.12% ID/g). Both single and double dose approaches significantly increased the tumor-to-muscle and tumor-to-blood ratios compared to the approach without the clearing agent. Especially, a 125-fold improvement of the tumor-to-blood ratio was achieved with a double dose of clearing agent [15]. The clear of mAb-TCO from circulation and nontumor tissues accelerated by the clearing agent exhibited important significance in PRIT due to the minimal normal tissue toxicity. Shah et al. applied the same clearing agent in a PRIT study by removing unbound mAb-TCO from the blood using the double dose approach. This study successfully demonstrated that pretargeted therapy using the clearing agent resulted in reduced tumor growth rates and improved survival with minimal normal tissue toxicity [41].

### 4.2. Influence Factors of Pharmacokinetic Properties of Radiolabeled Tz Derivatives

#### 4.2.1. Net Charge

It has been shown that the overall net charge of radiolabeled Tz derivatives influenced their clearing pathway. Several studies reported that the introduction of positive charge to a radiopharmaceutical can increase renal clearance and retention [54–56]. Similarly, a positive charge reduced the lipophilicity of the Tz radioligand and induced clearance through the kidneys, whereas Tz compounds with no charge were mainly excreted via the liver and intestines. In a pharmacokinetic relationship study including 25 different Tz derivatives radiolabeled with either Al[^18^F] or ^68^Ga, Meyer et al. [57] observed the different excretion pathways between ^68^Ga-NODA-Tz (renal excretion) and Al[^18^F]-NODA-Tz (hepatic and intestinal excretion). The charge difference between ^68^Ga-NODA-Tz in a charge of +1 and Al[^18^F]-NODA-Tz in a charge of 0 resulted in the different excretion pathway (Table 2). Similarly, compared with Al[^18^F]-NOTA-lysine-Tz (net charge: 0) with a primary hepatic and intestinal excretion, Al[^18^F]-NOTA-(lysine)_2_-Tz (net charge: +1) and Al[^18^F]-NOTA-(lysine)_3_-Tz (net charge: +2) clear quickly and primarily through the urinary tract.

As ^64^Cu-NOTA-Tz is eliminated slowly through the gastrointestinal pathway [26], Zeglis et al. [27] further created two novel Tz radioligands (^64^Cu-NOTA-PEG_7_-Tz and ^64^Cu-SarAr-Tz) with structural alterations geared at improving their pharmacokinetic profiles. ^64^Cu-SarAr-Tz not only changed the coordination environment from N_3_O_3_ to N6 but, more importantly, shifted the overall charge of the metal ligand complex from −1 (CuII-NOTA) to +2 (CuII-SarAr). Finally, ^64^Cu-SarAr-Tz clears quickly and primarily through the urinary tract, and ^64^Cu-NOTA-PEG_7_-Tz excreted through both the intestines and the kidneys. Additionally, the change of chelator from NOTA to SarAr may also play a role in the pharmacokinetic of ^64^Cu-Tz radioligands.

#### 4.2.2. Linker (Polypeptide and PEG) between Chelator and Tz Scaffold

The choice of polypeptide linker between the chelator and the Tz scaffold had an impact on the pharmacokinetic of radiolabeled Tz-derivatives. In the study conducted by García et al. [24], ^99m^Tc-HYNIC-PEG_4_-Tz was mainly eliminated via the hepatic pathway with 9.91 ± 0.97% ID/g and 23.35 ± 3.84% ID/g in the liver and intestines, respectively, 1 h after administration. However, ^99m^Tc-HYNIC-PEG_5_-polypeptide-Tz showed reduced gastrointestinal accumulation and increased the clearance through kidneys, with 81.92 ± 5.06% ID eliminated by urine after 1 h. Moreover, it has been previously reported that peptide renal uptake could be related to the presence of lysine residues, something that should be avoided in the rational sequence design of the linker [58, 59].

A large amount of literature demonstrated the ability of PEG (polyethylene glycol) linker to accelerate the clearance and lower the nontarget tissue uptake of radiopharmaceutical [60–62]. Zeglis et al. [27] found that the addition of the PEG_7_ linker (^64^Cu-NOTA-PEG_7_-Tz) improved the pharmacokinetics of ^64^Cu-NOTA-Tz. The pretargeted image distinctly showed that although both tracers showed rapid accumulation in the tumor tissues, ^64^Cu-NOTA-PEG_7_-Tz cleared significantly more quickly than ^64^Cu-NOTA-Tz (especially the clearance from digestive tract), clearly delineating the tumor tissue in PET images as early as 6 h after administration. Recently, another investigation reported that addition of a PEG linker improved *in vivo* pharmacokinetics of Tz derivatives for the *in vivo* pretargeted approach [44].

#### 4.2.3. Hydrophilicity

It is known that the Tz-scaffold part in the Tz-derivative radioligands is hydrophobic. Hydrophilic linkers and chelators should be considered in the synthetic design to improve the hydrophilicity of Tz-derivative radioligands. In the reported literature using pretargeted strategy, NOTA and DOTA were the most commonly used chelators for Tz derivatives because their carboxyl groups could significantly improve the hydrophilicity of the ultimate Tz-derivative compounds. For example, the radioligands ^64^Cu-NOTA-Tz and ^64^Cu-NOTA-PEG_7_-Tz were proved reasonably hydrophilic, with a logD of −2.54 ± 0.1 and −2.44 ± 0.08, respectively [27]. However, when the HYNIC was used as the chelator, the ^99m^Tc-HYNIC-Tz and ^99m^Tc-HYNIC-PEG_4_-Tz showed poor hydrophilicity, with a logD of −0.54 ± 0.07 and −0.62 ± 0.03, respectively. The author further added a polypeptide between the chelator and the Tz scaffold (^99m^Tc-HYNIC-PEG_5_-polypeptide-Tz) to increase the hydrophilicity to a logD of −1.05 ± 0.02 [23, 24]. Besides, Nichols et al. [5] and Devaraj et al. [29] synthesized a ^68^Ga- and ^18^F-labeled Tz-coated polymer based on a well-established hydrophilic aminodextran backbone, respectively.

In fact, pharmacokinetics observed for the Tz-derivative radioligands may be the result of the complex interplay of a variety of factors, including but not limited to charge, linker, hydrophilicity, protein binding affinity, and molecular structure. Ultimately, a preferable plasma half-life and excretion pathway should be considered when designing of Tz-derivative radioligand with all influencing factors considered. The plasma half-life of the Tz-derivative radioligands affected the tumor accumulation and was suggested to preferably exceed 10 min for good tumor accumulation in pretargeted experiments [57].

## 5. Summary and Perspective

As the singular combination of selectivity, orthogonality, and rapidity of the IEDDA reaction make it almost ideally suited for radiochemical applications, the IEDDA cycloaddition reaction has brought about a significant impact on the field of bioorthogonal radiopharmaceutical chemistry during the past 10 years. As we have discussed above, the IEDDA reaction has already been effectively employed in pretargeted nuclear imaging and radioimmunotherapy and showed a promising prospective. Yet despite these successful applications, some important obstacles remained to be addressed in this exciting field. For example, the syntheses of the Tz and TCO precursors are somewhat cumbersome. The stability and reactivity of either Tz or TCO are affected by several influence factors *in vivo* and *in vitro*. Additionally, the TCO tag modified mAbs applied in the pretargeted click methdology are generated through the use of secondary, chemically selective conjugation agents such as activated esters in TCO-NHS, which add an additional synthetic step and thus may reduce yield and even change its pharmacokinetics. There is a need for more radiolabeled probes with good pharmacokinetic and biodistribution profiles to be synthesized. Despite these obstacles, currently, the bioorthogonal IEDDA click chemistry has convincingly expanded to living mice. For human applications, the reagent concentrations will be much lower and reactivity and stability need to be higher than in mice. In addition, the optimized pretargeted systems should be evaluated with respect to sterility, toxicology, and immunogenicity [4]. In summary, we believe that the IEDDA cycloaddition reaction will have the potential to become central in the pretargeted nuclear imaging and radioimmunotherapy in the near future, crossing the bridge between fundamental and clinical research and ultimately benefitting patients at the bedside.

## Figures and Tables

**Table 1:** Overview of reported pretargeted nuclear imaging and radioimmunotherapy studies based on the IEDDA click chemistry.

Author (year)	Xenograft model	Target	Immunoconjugate	Tz radioligand	Others
Rossin et al. (2010) [13]	LS174T colon cancer	TAG-72	CC49-PEG_12_-benzamide-TCO	^111^In-DOTA-PEG_11_-bispyridyl Tz	—
Rossin et al. (2013) [14]	LS174T colon cancer	TAG-72	CC49-benzamide-TCO or CC49-PEG_12_-benzamide-TCO	^111^In-DOTA-PEG_11_-bispyridyl Tz	Axially substituted TCO with bulky linker and no PEG linker compared with Reference [7]
Rossin et al. (2013) [15]	LS174T colon cancer	TAG-72	CC49-benzamide-TCO	^111^In-DOTA-PEG_11_-bispyridyl Tz	Clearing agent and no PEG linker compared with Reference [7]
Rossin et al. (2014) [16]	LS174T colon cancer	TAG-72	CC49-acetamide-TCO	^111^In-DOTA-PEG_11_-bispyridyl Tz	Acetamide linker between CC49 and TCO compared with benzamide linker in Reference [7–9]
Altai et al. (2016) [20]	SKOV-3 ovarian cancer	HER2	Z_2395_ (affibody)-PEG_4_-TCO	^111^In-DOTA-PEG_10_-bispyridyl Tz	—
van Duijnhoven et al. (2015) [21]	LS174T colon cancer	TAG-72	AVP04-07 (diabody)-benzamide-TCO	^111^In-DOTA-PEG_11_-bispyridyl Tz	—
García et al. (2016) [23]	LS174T colon cancer	TAG-72	CC49-TCO	^99m^Tc-HYNIC-Tz	—
García et al. (2018) [24]	B16-F10 melanoma	VEGF	Bevacizumab-TCO	^99m^Tc-HYNIC-PEG4-Tz^99m^Tc-HYNIC-PEG_5_-polypeptide-Tz	Polypeptide: Gly-Arg-Glu-Arg-Glu-Lys
Yazdani et al. (2016) [25]	—	Bone	BP (bisphosphonate)-TCO	^99m^Tc-Tz complex	—
Zeglis et al. (2013) [26]	SW1222 colorectal cancer	HuA33	A33-TCO	^64^Cu-NOTA-Tz	—
Zeglis et al. (2015) [27]	SW1222 colorectal cancer	HuA33	A33-TCO	^64^Cu-NOTA-PEG_7_-Tz^64^Cu-SarAr-Tz	^64^Cu-SarAr-Tz net charge (+2)
Evans et al. (2014) [28]	A431 cutaneous squamous cancer	EGFR	Cetuximab-PEG_4_-TCO	^68^Ga-DOTA-Tz	—
Nichols et al. (2014) [5]	LS174 T colon cancer	HuA33	A33-TCO	^68^Ga-DTPA aminodextran Tz-coated polymer	Aminodextran polymer: 16 kDa
Devaraj et al. (2012) [29]	LS174 T colon cancer	HuA33	A33-TCO	^18^F aminodextran Tz-coated polymer (^18^F-PMT10 and PMT40)	Aminodextran polymer: 10 and 40 kDa
Meyer et al. (2016) [34]	BxPC3 pancreatic cancer	CA19.9	5B1-TCO	Al[^18^F]-NOTA-PEG_11_-Tz	—
Shi et al. (2018) [35]	HCT116 colon cancer	EGFR	Cetuximab-Tz and panitumumab-Tz	Al[^18^F]-NOTA-TD (a Reppe anhydride derivative)	An inverse strategy and replaced TCO with a Reppe anhydride derivative
Denk et al. (2016) [36]	—	Lung	MSNs-TCO and MSNs-s-TCO	^11^C-Tz	First^11^C-labeled Tz
Keinänen et al. (2017) [37]	—	Spleen and liver	NPs-TCO	^18^F-Tz	—
Keinänen et al. (2017) [38]	A431 epidermoid carcinoma BT-474 ductal carcinoma	EGFR HER2	Cetuximab-TCO and trastuzumab-TCO	^18^F-Tz	—
Houghton et al. (2017) [39]	BxPC3 pancreatic cancer	CA19.9	5B1-TCO	^177^Lu-DOTA-PEG_7_-Tz^177^Lu-CHX-A”-DTPA-PEG_7_-Tz	—
Membreno et al. (2018) [40]	SW1222 colorectal cancer	HuA33	A33-TCO	^177^Lu-DOTA-PEG_7_-Tz	—
Shah et al. (2017) [41]	LS174 T colon cancer	TAG-72	CC49-TCO	^212^Pb-DOTA-PEG_11_-bispyridyl Tz	—

**Table 2:** Comparison of the molecular net charge and the primary elimination route for different radiolabeled Tz derivatives.

Radiolabeled Tz derivatives	Molecular net charge	Primary elimination route
Al[^18^F]-NODA-Tz	0	Hepatic and intestinal excretion
^68^Ga-NODA-Tz	+1	Renal excretion
Al[^18^F]-NOTA-lysine-Tz	0	Hepatic and intestinal excretion
Al[^18^F]-NOTA-(lysine)_2_-Tz	+1	Renal excretion
Al[^18^F]-NOTA-(lysine)_3_-Tz	+2	Renal excretion
^64^Cu-NOTA-Tz	−1	Hepatic and intestinal excretion
^64^Cu-NOTA-PEG_7_-Tz	−1	Hepatic and intestinal excretion
^64^Cu-SarAr-Tz	+2	Renal excretion
